# Assessment of losses to the local population due to restrictions on their ownership rights to land and property assets: The case of the Tunkinsky National Park, Russia

**DOI:** 10.1371/journal.pone.0251383

**Published:** 2021-05-10

**Authors:** Lyudmila Maksanova, Taisiya Bardakhanova, Natalia Lubsanova, Darima Budaeva, Arnold Tulokhonov

**Affiliations:** Baikal Institute of Nature Management Siberian Branch of the Russian Academy of Sciences, Ulan-Ude, Republic Buryatia, Russia; Szechenyi Istvan University, HUNGARY

## Abstract

The impact of protected areas on local communities is the subject of intense discussions as part of the implementation of the global ecosystem protection agenda. Conflicts between the interests of environmental protection and the needs of socio-economic development become particularly acute when large areas of land are taken out of economic circulation as a result of organizing protected areas. In this case, there is an urgent need for detailed and reliable information about the social impacts of such land withdrawal on the well-being of the local population. An analysis of the methodological approaches widely presented in the literature, used to assess the social impact of protected areas, testifies to the insufficiency of completed and practically applicable methodological guidelines for the areas with significant restrictions for people who form part of the protected landscape. In this study, we understand the cost estimate of the social impact of national parks on the local population as a quantitative calculation of the losses due to restrictions on their ownership rights to land and property assets. The methodological approach consists in considering the category of losses as a sum total of the actual damage and lost profits. The assessment algorithm includes three stages: systematization of social impacts on citizens, development of indicators and data collection, and calculation of actual damage to the population and lost profits. The assessment is performed using the example of the Tunkinsky National Park located in the Tunkinsky municipal district of the Republic of Buryatia, a region of the Russian Federation, where there are 14 rural settlements with a population of more than 20,000 people. The results of the calculations show that the losses of the rural population due to legal restrictions on the registration of land dealings amount to 170.4 million USD. Taking into account the potential amount of administrative fines and the value of property subject to demolition, the losses amount to 239.2 million USD. It is more than an order of magnitude greater than the amount of own revenues of the Tunkinsky municipal district in 2011–2019. The results obtained demonstrate the real picture of the impact of restrictions on the rights of local people to land within the boundaries of national parks and are useful for developing measures to account for their interests and include protected areas in the socio-economic development of regions. The methodological approach developed by the authors can be used in other national parks, where it is necessary to optimize the policy of improving land use for local residents.

## Introduction

The creation of a network of protected areas is a critical tool in promoting the values of conservation of biodiversity, natural resources, and ecosystem services. Originally conceived to conserve unique landscapes and wildlife, protected areas are undergoing a transformation of approaches to further development under the Durban Agreement (2003) [1]. In line with these transformations, the task of shaping the interaction of people with nature in the landscapes of which they are a part has not lost its relevance [2]. Therefore, assessments of the impact of protected areas on the well-being of local residents have become increasingly common, with both positive and negative effects [3]. Oldekop et al. (2016), based on a study of 171 papers on 160 terrestrial and marine protected areas across six continents, found that protected areas that empower local communities and/or bring socio-economic benefits to local communities are more likely to achieve positive outcomes in biodiversity conservation and climate change mitigation. At the same time, large protected areas may have greater social and economic impacts for people than small ones [4].

In some countries, protected areas contribute positively to poverty reduction, as in Ethiopia, where households within and adjacent to three national parks have higher incomes than those living outside them [5].

At the same time, there are examples of restrictions imposed by the government on the interests of the local population in very sensitive natural areas or areas of traditional nature management that inevitably lead to social, environmental, and economic conflicts [6, 7]. The prohibition of access to natural resources can cause discontent and lack of support for the conservation goals among the local population [8, 9].

In continuation of the ongoing debate about the importance of considering human well-being in biodiversity conservation [10], new evidence is emerging in various countries about the effects of conservation policies on local residents living within protected areas [11], including in Russia [12].

Russian protected areas have not traditionally been considered in the context of fulfilling the objectives of development or improving the well-being of local residents [13]. Decisions to create protected areas, as elsewhere in the world, were made primarily in order to ensure biodiversity conservation with the adoption of overly strict legal restrictions for people [14]. In line with the Durban Agreement, researchers increasingly recognize the need to develop equitable policies for the creation and maintenance of protected areas, taking into account the protection of the rights and interests of people [9, 15]. The development of such policies can rely on social impact assessment of protected areas, which is understood as an assessment of the likely positive and negative impacts of development activities that may affect economic, social, cultural, civil, and political rights, as well as community quality of life, as measured by various socio-economic indicators [16]. However, despite the importance of assessing social impacts, they remain understudied [17]. There is a need to develop methodological approaches to measure the social impacts of protected areas, taking into account the types of protected areas and the factors affecting the level of social impacts, as well as due to the lack of a standard tool to assess the social impact.

In this paper, the authors focus on the development of a methodology to quantify the social impacts arising from the restriction (impairment) of property rights on land and property assets of local residents, as well as the rights to measures of their social support. This assessment is based on the example of a specific type of protected area—a national park, the creation and functioning of which significantly impacts the population living within its borders. The results of the study demonstrate a reliable quantitative assessment of the social impacts on the local population living within the borders of the Tunkinsky National Park (Russia) and can be used in the preparation of management decisions. The authors hope to contribute to the development of the methodology for assessing the social impacts of protected areas.

## Literature review

The principle that protected areas should not cause harm to local populations is enshrined in the Durban Agreement adopted at the World Congress on Protected Areas in 2003. Social impacts are the main factor influencing the social acceptability of conservation activities [17]. Under the social impacts the authors consider the planned, or unexpected, spontaneous results of transformations in society, affecting the social relations of groups of people and individuals. In the case of protected areas, these impacts can encompass many aspects of human well-being, which, according to the Millennium Ecosystem Assessment guidance document, are centered around the basic material needs for normal life, health, normal social relationships, safety, and freedom of choice and action [18]. Each of these five components of well-being includes several subcategories described by a broad set of indicators, the identification of which is a complex task in social impact assessment methodology [3]. Jones, N. et al. (2017), upon reviewing case studies and theoretical discussions in the biodiversity conservation and social impact assessment literature, identified the following major social impacts on people that the creation of protected areas brings about: poverty, health, displacement, redistribution of power, and human rights [17].

The literature provides quite convincing evidence that protected areas can help reduce poverty in local communities [19], as well as examples of how in developing countries a large part of the population depends on natural resources for their livelihood, so households within national park boundaries lose income directly or indirectly (losses in beekeeping, in grazing due to attacks by wild animals, in using firewood, etc.) [20]. The late 20th century concept of “New Conservation”, which recognized the need to compensate “the community” for the direct and indirect costs of conserving natural resources [21, 22], including from funds generated by ecotourism [23], held promise for reducing poverty in communities adjacent to protected areas and for compensating rural residents for lost profits incurred due to conservation policies and the increased costs of living near a protected area. However, a case study of two national parks in Tanzania found that the benefits of compensating protected area populations for resource constraints were modest and did not meet the needs of predominantly poor men and women [24].

Regarding the health and safety impacts of protected areas, a systematic review commissioned by the Global Environment Facility indicated that opinions on local health are mostly negative, and quantitative studies of the health and safety impacts of protected areas are notable for their absence [25].

Forced or involuntary displacement of people is one of the most negative social impacts of protected areas. An example of a study of the effects of relocating indigenous people from a national park in Honduras shows that park rules and policies led to changes in access to land for subsistence needs and the increased intensity of land use, which, in turn, racked up production costs and contributed to changes in income-generating activities and an increased role of wage labor as a source of subsistence income [26].

The introduction of a new management structure that implements new rules regarding the development of the area and natural resources is a key theme in studies on the impact of protected areas on the well-being of local people [17, 25, 27, 28]. And this, according to the authors, is just the beginning of a chain of social consequences of the adoption of conservation measures.

Researchers recognize that the creation and functioning of protected areas can have a significant impact on human rights in local settlements located within the boundaries of protected areas [14, 25]. However, historically, impact assessment practices have not explicitly considered human rights [29], and this aspect is not widely represented in assessment practices [17]. The range of human rights is quite significant, including rights to land, water, natural resources, education, labor, recreation, etc. At the same time, the right of land ownership is one of the basic institutions determining the nature of social and economic relations, especially in rural areas. Therefore, the infringement (limitation) of property rights to land and property assets is among the most resonant social impacts. Proof of this is the example of land ownership disputes of indigenous people in Australia, both between local residents and the administration of protected areas, and among local residents from different groups living near the Purnululu National Park [25].

In Russia, land dealings in national parks has been limited. Consequently, the local population living in national parks for many years could not fulfill the right to have private ownership of land and to exercise local self-government, since any issues of economic activity must be decided by the national park administration. Thus, the problem of restricting citizens’ rights to land exists in 27 national parks across Russia, where there are about 923 settlements with a total population of 371,000 people [30]. It is necessary to note that the recently adopted amendments to the Federal Law 505-FZ “On Specially Protected Natural Areas” dated December 30, 2020, provide the possibility of privatization and land dealings in settlements located in protected areas, but only in those whose boundaries are entered in the Unified State Register of Real Estate. The issues of assessing the social impacts of legal restrictions on the livelihood of citizens and economic activities within the boundaries of protected areas are insufficiently studied. The available publications only document or describe the existing negative impacts of human activity within the borders of national parks [13, 14].

In the international social impact assessment community, social impact assessment refers to the process of analyzing and managing the intended and unintended social consequences, both positive and negative, of planned interventions (policies, programs, plans, or projects) [31]. Since the first systematic review of empirical evidence of human well-being impacts arising from the creation and maintenance of terrestrial protected areas [25], there has been a methodological search for improving approaches to assess the social impacts of protected areas [31, 32]. These studies critically analyze existing practices; examine the most commonly used methodologies, which are applied in original and adapted formats; draw attention to the extreme complexity of determining social impact indicators; emphasize impact factors, choice of data collection method, and sampling period and duration; consider a combination of quantitative and qualitative data, etc. [3, 17, 30].

Jones, N. et al. believe that despite the increase in the number of studies devoted to assessing the social impact of protected areas, there is still no official protocol or generally accepted methodological tools [17]. Therefore, in the absence of unified methodological approaches to the assessment of the social impact of protected areas, of particular value are methodological recommendations to improve the assessment procedure. Thus, Pullin A. et al. when measuring the impacts on the welfare of the local population recommend to take into account the differences of terrestrial protected areas by their status, management, and goals. For example, some local communities may be located in a protected area, others—in buffer zones around the borders of protected areas, and still others—in more remote areas [25]. De Lange et al. identify three main components of assessment: selection of indicators, research design, and data collection [3]. The recommendations of Jones, N. et. al. regarding the development of a methodology that could be applied in different areas allowing the assessment of social impacts and simultaneously comparing them between different protected areas are important [17].

Thus, a review of the literature testifies to the urgency of assessing the social impact of protected areas and the complexity of developing methodological approaches due to the multifaceted nature of the subject of research.

## Methods and materials

### Research methodology

This study is based on the methodology of assessing the impacts of introducing environmental restrictions in the Baikal Natural Area in order to protect the unique ecosystem of Lake Baikal, the largest reservoir of the world’s fresh water and a World Natural Heritage site. Federal Law 94-FZ “On the Protection of Lake Baikal” dated May 01, 1999, introduced strict ecological restrictions on the scale and nature of the use of natural resources, as well as high-level requirements for emissions and discharges of pollutants. The development and carrying out of environmental measures entailed higher capital and operating costs, direct production losses, and lost profits in the economy, in the entire drainage basin of Lake Baikal. The methodological approach to the assessment of environmentally conditioned additional costs and lost profits consisted in determining the essence of the environmental constraint and its scope and identifying changes in the conditions of production and daily activities that were likely to occur as a result of environmental constraints, as well as in revealing quantitative relationships between such changes and the costs. The methodological approach was tested on the example of the Republic of Buryatia and the Tunkinsky National Park [33].

In this article, the subject of the study is a quantitative assessment of the social impacts of legal restrictions on land dealings for the population, by which we mean the cost expression of losses resulting from the infringement of (or failure to respect) the rights of citizens living in settlements located within the boundaries of a protected area. In this case, we adhere to the legal interpretation of losses (Clause 2 of Article 15 of the Civil Code of the Russian Federation). Losses are understood as the totality of actual damage and lost profits of persons (citizens) whose right has been infringed. In turn, the actual damage is the incurred or future expenses to restore the infringed right, as well as a loss or damage to property. Lost profits are lost income, which the person would have received under normal conditions of civil commercial transactions, if this person’s right was not infringed. Since we are talking about the supposed material addition to the property of the victim, which would have occurred in the normal course of events if the right had not been infringed, it should be taken into account that the calculation of lost profits is usually approximate and has a probabilistic nature. Table 1 presents the types of public losses resulting from the infringement of (or failure to respect) the rights of citizens living in communities located within the boundaries of protected areas due to legal restrictions on land registration.

**Table 1:** Losses of the population living within the boundaries of protected areas as a result of legal restrictions on registration of land dealings.

Types of losses	Indicators and definitions
Actual damage	
**Costs incurred**	Capital costs for the construction of real estate objects or infrastructure (AD_1_), for the demolition/relocation of which orders have been issued
	The amount of paid administrative fines for the lack of title documents for land lots, (AD_2_)
	Costs incurred for environmental impact assessment, coordination of social and economic activities with the RF Ministry of Natural Resources, (AD_3_)
	Loss of personal income from personal subsidiary lots, (AD_4_)
**Future costs to restore the infringed right**	Costs of forced relocation, (AD_5_)
	Expenses for the purchase of land and construction to replace the lost property, (AD_6_)
	Potential costs for environmental impact assessment, coordination of social and economic activities with the RF Ministry of Natural Resources, (AD_7_)
**Loss or damage to property**	The cost of full or partial loss of property due to relocation from protected areas, (AD_8_)
	Material losses of the people due to inability to sell land lots (RD_9_)
**Social support losses**	Material losses of the people incurred as a result of the failure to provide social support, (AD_9_)
Lost profits	
**Lost income of the population**	Due to the reservation of funds for construction (LP_1_)
	Due to the diversion of funds for relocation (LP_2_)
	Due to the diversion of funds for fines (LP_3_)

Source: Compiled by the authors.

The algorithm of the cost estimation of losses developed by the authors of this paper includes three stages.

Stage 1 “Systematization of social impacts on citizens” identifies legal restrictions on livelihoods and economic activities within the boundaries of national parks, as well as the emerging social impacts on citizens. In this study, legal obstacles to the registration of land dealings are considered as the most significant constraint.

Stage 2 “Development of indicators and data collection” defines a set of baseline indicators that characterize (describe) the impact of the above-described constraints on living conditions, economic activity settings, and the arrangements for the delivery of social commitments to the local population. The data collection takes into account the principles of information availability, as well as the period since the registration of the land title was denied.

Calculations of the actual damage and lost profits are performed at Stage 3 “Quantitative assessment of material losses incurred due to legal restrictions on the registration of land dealings”:

1) The assessment of actual damage to the population (*ADpop*) is carried out by a direct accounting method based on the cost approach using the following formula:

$${AD}_{pop}=\sum_{i=1}^{10}{AD}_{i}$$ (1)

The formulas for calculating the individual components of actual damage are presented in Table 2. The need for the already incurred and future expenses and their estimated amount must be confirmed by a reasonable calculation based on costing and other evidence. In case of loss of property, the value of the lost property is determined after deduction of depreciation. In case of damage to property, the amount of depreciation or expenses for repairing the damage are determined.

**Table 2:** Formulas for calculating the initial indicators to quantify actual damage.

Indicators	Calculation formulas
Capital costs for the construction of real estate objects or infrastructure (*AD*_1_), for the demolition/relocation of which orders have been issued	*AD*_1_ = *N*_demolition_*S_build_*P_*build*_, where: (4)
	*N*_*demolition*_—the number of orders or court decisions to demolish/relocate residential buildings located in protected areas, pcs.
	*S*_*build*_—average floor space of one apartment in private housing, sq. m;
	*P*_*build*_—average actual construction cost of 1 sq. m., thousand rubles.
Potential capital costs of construction of real estate objects or infrastructure (*AD*′_1_), for demolition/relocation of which orders were issued	${AD\prime }_{1}=N_{land}^{ind}*S_{build}*P_{build}$, where: (5)
	$N_{land}^{ind}$—the number of unregistered land lots for individual housing development located in protected areas, pcs.
The amount of paid administrative fines for the lack of title documents for land lots (*AD*_2_)	*AD*_2_ = *N*_penalty_*R_*penalty*_, where: (6)
	*N*_*penalty*_—the number of citizens brought to administrative liability for the lack of registered rights to land lots, residential houses, and premises located in protected areas, pers.
	*R*_*penalty*_—the amount of fine for the lack of title documents for land lots, thousand rubles.
Potential amount of administrative fines for the lack of title documents for land lots (*AD*′_2_)	${AD\prime }_{2}=N_{land}^{ind}*R_{penalty}$ (7)
Loss of personal income from personal subsidiary lots, (*AD*_4_)	${AD}_{4}=N_{left}^{work}*{AP}_{priv}$, where: (8)
	$N_{left}^{work}$—the number of people of working age who left protected areas, pers.
	*AP*_*priv*_—production of agricultural products in personal subsidiary lots per 1 person.
Costs of forced relocation (*AD*_5_)	*AD*_5_ = *N*_left_*P_*fare*_, where: (9)
	*N*_*left*_—the number of people who left protected areas, pers.
	*P*_*fare*_—cost of travel, thousand rubles.
Expenses for the purchase of land and construction to replace the lost property (*AD*_6_)	${AD}_{6}=N_{left}/\bar{S_{n}}*{S^{\prime }}_{land}^{ind}*{P\prime }_{land}$, where: (10)
	$\bar{S_{n}}$—average household size.
	${S^{\prime }}_{land}^{ind}$—average area of land lots for individual residential development at a new location, sq. m.
	*P*′_*land*_—a specific indicator of the cadastral value of land for low-rise residential development, including individual residential development, at the new location, rubles per sq. m.
The cost of full or partial loss of property due to relocation from protected areas (*AD*_8_)	${AD}_{8}=N_{left}/\bar{S_{n}}*S_{build}*P_{build}$, where: (11)
	$S_{land}^{ind}$—average area of land lots for individual residential development in protected areas, sq. m.
	*P*_*land*_—a specific indicator of the cadastral value of land for low-rise residential development, including individual residential development, in protected areas, rubles per sq. m.
Material losses of the people due to inability to sell land lots (*AD*_9_)	$AD_{9}=N_{land}^{ind}*S_{land}^{ind}*P_{land}$, where: (12)
	$N_{land}^{ind}$—the number of land lots for individual residential development with unregistered titles, located in protected areas.
Material losses of the people incurred as a result of the failure to provide social support (*AD*_10_)	${AD}_{10}=N_{family}*S_{land}^{family}*P_{land}$, where: (13)
	*N*_*family*_—the number of multi-child families without registered land lots.
	$S_{land}^{family}$—the minimum size of the land lot provided to a multi-child family, sq. m.

Source: Compiled by the authors.

2) Calculation of the amount of lost profit (*LP*_*pop*_) is performed using the rate of return on invested capital (*i*):

$${LP}_{pop}=\sum_{j=1}^{3}{LP}_{j}$$ (2)

The individual components of lost profits can be calculated using to the following formula:

$${LP}_{j}={AD}_{j}*({\left(1+i\right)}^{t}-1),$$ (3)

where:

*AD*_*j*_–losses of the j-th type, as a result of which no income could be received;

*t*—the period during which income could not be received as a result of the diversion of funds.

### Study area: Tunkinsky National Park

There are 56 national parks in Russia. In 27 of them there are 923 communities. Russian law allows settlements with people living in them to be located in national parks. However, the rights of people living in national parks are significantly limited, since land lots and natural resources located within the boundaries of national parks are owned by the federal government, with the exception of land lots with previous private property rights.

It is prohibited to provide land lots in national parks for gardening and horticulture, the construction of private garages or houses. It is also prohibited to build sports facilities that are classified as capital facility projects and the associated engineering and transport infrastructure. All this leads to a significant restriction of the rights of people living in national parks.

Tunkinsky National Park is located in the southwestern part of the Republic of Buryatia, a region of the Russian Federation, at a distance of about 50 km from the world-famous Lake Baikal. The territory of the park stretches from west to east for 190 km with the following coordinates: from north to south between 52°05`and 51°05 ’ north latitude and from west to east between 100°45`and 103°45`east longitude. The southwestern border of the national park coincides with the state border of Russia with Mongolia.

Established in 1991, this park is one of the largest national parks in Russia and meets all criteria for the organization of protected areas, such as preservation of natural complexes, uniqueness of biological resources, landscape and biological diversity, high recreational suitability, and historical and cultural value.

Unlike all other Russian national parks, the Tunkinsky National Park is characterized by two distinctive features. Firstly, this park completely covers the whole territory of the Tunkinsky municipal district. Secondly, in the east, a portion of the park is included into the Central Ecological Zone of the Baikal Natural Area, where strict environmental restrictions are in force. The total area of the park (11,186.62 km^2^) is divided into the land of the National Park (90%) and the land of other owners (10%).

In terms of nature, the park covers “the Tunka branch of the inter-mountain basins belonging to the southwestern Baikal region, and stretches from the south-western tip of Lake Baikal in a sub-latitudinal direction for more than 200 km. From the north, the territory is bounded by the Tunkinsky mountains, and from the south—by the Khamar-Daban range”. In the park, there are plants of steppe landscapes and mountain alpine meadows, as well as various species of rare and endangered plants included in the Red Book of Russia. The fauna is characterized by the animals of the steppe, taiga, and rocky mountains. A unique animal living in the park is the snow leopard, which is listed in the Red List of the International Union for Conservation of Nature as an endangered species.

A differentiated regime of conservation and land use has been established in the park. According to this regime, the following zones were allocated in the park: nature reserve, specially protected zone, recreational zone, and economic zone. There are 35 communities located in the economic zone, united in 14 rural settlements.

Historically, when the Tunkinsky National Park was created, in addition to all state forestry lands and the state reserve land, it included agricultural lands, lands of settlements, and lands of other land users without withdrawal from economic use, which was reflected in Resolution No. 282 of the RSFSR Council of Ministers dated May 27, 1991, and Resolution No. 353 of the Council of Ministers of the Buryat Soviet Socialist Republic, dated December 31, 1991. However, after the adoption of the Federal Law “On Specially Protected Natural Areas” in 1995, which established that protected areas could also include land lots fully or partially withdrawn from economic use, appropriate changes were not introduced into the legal documents on the establishment of the park.

**Fig 1:**
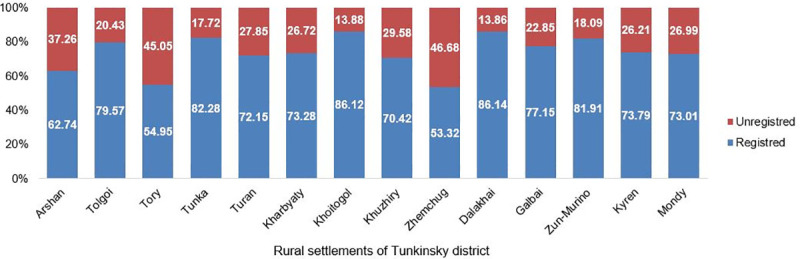
Share of unregistered land lots in the total number of lands lots of rural settlements of Tunkinsky district. (Source: Compiled by authors based on data Administration of the Tunkinsky municipal district of the Republic of Buryatia (S1 Table).

In 2011–2012, land dealings began to be restricted. The registration of ownership rights to agricultural land lots and lands of settlements was discontinued. The proportion of unregistered land lots by the end of 2020 in the whole municipal district was 32.7%. In rural settlements the share of unregistered land lots varies from 13.9% to 46.9% (Fig 1).

As a result, residents are limited in their rights to exclusive ownership of land, to use it, to transfer it by donation, bequest or inheritance, to transfer it for use to others, to receive income from the use by others, to guarantee property. The ban on the registration of property rights to land hindered the social security of multi-child families and young professionals, which in turn led to the induced relocation of citizens. Thus, the number of the local population has decreased by 9% since 2012, from 22,084 to 20,106 [34]. The “land” barrier had a negative impact on entrepreneurial activity in the municipality. Between 2012 and 2019, the number of small businesses in the Tunkinsky municipal district halved, and in 2019, the district’s share in the regional structure by this indicator was only 0.3%. Problems with land registration, unemployment, lack of career prospects and favorable conditions for children’s development, etc. contributed to the migration outflow of the district’s population (S2 Table). Thus, in 2019, out of 1,074 people who left the municipality, 66.1% were people of working age (710 people) and 20.8% were under working age (223 people). The decline in the population and entrepreneurial activity also negatively influences the dynamics of the municipality’s own revenues, which have tended to decrease since 2015 (S3 Table).

### Data collection

According to the developed methodology, data collection relies on official statistical data and materials presented in regulatory documents and reports of public authorities, the administration of the Tunkinsky municipal district, and the directorate of the Tunkinsky National Park. Table 3 presents baseline indicators for calculating the actual damage to the population. The amount of lost profits is estimated on the basis of the values of the indicators characterizing the actual damage to the residents of the Tunkinsky municipal district who have no registered rights to land lots.

**Table 3:** Baseline indicators for calculating actual damage to residents of the Tunkinsky district whose rights to land lots are not registered.

	Indicator	Definition	Value	Source
1.	Number of unregistered land lots, unit	*N*_*land*_	11,522	Administration of the Tunkinsky municipal district of the Republic of Buryatia
2.	Number of unregistered land lots for individual housing development, unit	$N_{land}^{ind}$	1,502	Administration of the Tunkinsky municipal district of the Republic of Buryatia
3.	Number of multi-child families without land lots, unit	*N*_*family*_	295	Administration of the Tunkinsky municipal district of the Republic of Buryatia
4.	Number of citizens brought to administrative liability for the lack of registered rights to land lots and property, person	*N*_*penalty*_	107	Office of the Federal Service for State Registration, Cadaster and Cartography in the Republic of Buryatia
5.	Number of orders or court decisions to demolish/relocate residential buildings	*N*_*demolition*_	0	Administration of the Tunkinsky municipal district of the Republic of Buryatia
6.	Number of people who left the Tunkinsky municipality during the period of restrictions, person	*N*_*left*_	9,125	Database of municipalities of the Republic of Buryatia https://rosstat.gov.ru/dbscripts/munst/munst81/DBInet.cgi#1
7.	Including people of working age, person	$N_{left}^{work}$	6,032	Database of municipalities of the Republic of Buryatia https://rosstat.gov.ru/dbscripts/munst/munst81/DBInet.cgi#1
8.	Average area of land lots for individual residential development in the Tunkinsky municipality, sq. m.	$S_{land}^{ind}$	1,000	Construction and land use regulations for settlements in the Tunkinsky municipality
9.	Average area of land lots for individual housing development in Ulan-Ude, sq. m.	${S\prime }_{land}^{ind}$	700	Construction and land use regulations of the Ulan-Ude urban district
10.	Specific indicator of the cadastral value of land for low-rise residential development including individual residential development in the Tunkinsky municipality, thousand rubles	*P*_*land*_	0.14231	Resolution of the Government of the Republic of Buryatia No. 2 “On approving the results of determining the cadastral value of lands of settlements in the Republic of Buryatia (as amended on June 05, 2020)” dated January 13, 2016
11.	Average cost of a land lot in Ulan-Ude, thousand rubles/sq. m.	*P*′_*land*_	0.50556	Resolution of the Government of the Republic of Buryatia No. 2 “On approving the results of determining the cadastral value of lands of settlements in the Republic of Buryatia (as amended on June 05, 2020)” dated January 13, 2016
12.	Average area of an apartment in individual residential development in the Republic of Buryatia in 2019, sq. m.	*S*_*build*_	86.6	Press release “On the commissioning of housing and social facilities in the Republic of Buryatia in 2019” https://burstat.gks.ru/currentevents/document/81152
13.	The average actual cost of construction of 1 sq. m. in the Republic of Buryatia in 2019, thousand rubles	*P*_*build*_	38.061	Press release “On the commissioning of housing and social facilities in the Republic of Buryatia in 2019” https://burstat.gks.ru/currentevents/document/81152
14.	The amount of fine for the lack of title documents for land lots, thousand rubles	*R*_*penalty*_	5	Article 7.1. of the Code of the Russian Federation on Administrative Offenses, dated December 30, 2001, No. 195-FZ (edited on December 30, 2020) http://www.consultant.ru/document/cons_doc_LAW_34661/
15.	Travel cost to Ulan-Ude, thousand rubles	*P*_*fare*_	0.9	https://03.avtovokzal-on-line.ru (retrieved on 12.12.2020)
16.	Average household size in rural settlements of the Republic of Buryatia, person	$\bar{S_{n}}$	3	Results of the 2010 All-Russian Population Census https://www.gks.ru/free_doc/new_site/perepis2010/croc/Documents/Vol6/pub-06-02.xlsx
17.	Production of agricultural products in personal subsidiary lots per 1 person	*AP*_*priv*_	26,882	Calculated based on the data of the All-Russian Agricultural Census of 2016 https://burstat.gks.ru/vshp2016 and the database of municipalities of the Republic of Buryatia https://rosstat.gov.ru/dbscripts/munst/munst81/DBInet.cgi#1
18.	Minimum size of the land lot provided to a multi-child family, sq. m.	$S_{land}^{family}$	400	Law of the Republic of Buryatia No. 115-III “On providing free land lots owned by the state and municipalities” dated October 16, 2002

Source: Compiled by the authors.

## Results and discussion

According to the data provided by the Tunkinsky District Administration, during the analyzed period from 2011 to the present time, no orders or court decisions have been issued regarding the demolition or relocation of residential buildings located in the Tunkinsky National Park. However, in fact, in accordance with existing legislation, all real estate property located on land lots that have no duly registered titles in the Tunkinsky National Park is subject to demolition or relocation. The potential amount of capital costs for the construction of real estate property (*AD*′_1_), for the demolition/relocation of which orders were issued, was determined based on the number of land lots for individual housing development without registered rights located in the Tunkinsky National Park, the average floor space of one apartment in individual housing development, and the average actual construction cost of 1 square meter of housing (Table 3). Capital construction costs were estimated at 4,950,716.07 thousand rubles:

$${AD\prime }_{1}=1,502*86,6\;sq.m.*38,061\;thous.rub./sq.m.=4,950,7116.07\;thous.rub.$$

The amount of administrative fines paid for the lack of title documents for land lots was 535 thousand rubles (*AD*_2_). It was calculated based on the total number of citizens brought to administrative liability for the lack of registered rights to land lots, houses, and premises located in the Tunkinsky National Park, and the minimum fine for individuals (Table 3).

$${AD}_{2}=5\;thous.rub.*107\;pers.=535\;thous.rub.$$

It should be taken into account that all residents living on land lots with unregistered rights located in the Tunkinsky National Park may be subjected to administrative liability. Therefore, the potential amount of administrative fines for the lack of title documents for land lots is 7,510 thousand rubles.

$${A\prime D}_{2}=5\;thous.rub.*1,502\;pers.=7,510\;thous.rub.$$

The amount of incurred and future expenses for environmental expertise and the coordination of activities with the Ministry of Natural Resources and Environment of the Russian Federation was not calculated (*AD*_3_, *AD*_7_), because the environmental expertise of the design documentation for individual housing development is not carried out, and the issuance of construction and reconstruction permits for the capital construction projects planned within the borders of protected areas of federal importance, as well as permits for commissioning these objects, is done without charging fees.

Taking into account the number of people of working age who left the Tunkinsky municipality in 2011–2019 and the data on the volume of agricultural production in private subsidiary lots per 1 person, the lost income of the population from private subsidiary lots (*AD*_4_) (Table 3) was as follows:

$${AD}_{4}=6,032\;pers.*26,882\;thous.rub.=162,152,2\;thous.rub.$$

Forced relocation costs (*AD*_5_) were determined based on the cost of travel to Ulan-Ude and the number of people who left the Tunkinsky municipality in 2011–2019:

$${AD}_{5}=9,125\;pers.*0,9\;thous.rub.=8,212.5\;thous.rub.$$

Land acquisition and construction costs to replace lost property (*AD*_6_) were estimated based on the cost and average area of land lots in Ulan-Ude:

$${AD}_{6}=9,125\;pers./3\;pers.*700\;sq.m.*0.50556\;thous.rub./sq.m.=1,076,539.46\;thous.rub.$$

The cost of complete or partial loss of property due to relocation from the protected area (*AD*_8_) was estimated at 10,026,683.27 thousand rubles.

Material losses of the people due to their inability to sell land lots (*AD*_9_) were determined according to the data presented in Table 3. The value of this type of losses to the population was 213,749.62 thousand rubles.

The assessment of the lost revenues of the population incurred due to inability to provide social support (*AD*_10_) was carried out for the category of multi-child families. According to the regional legislation, multi-child families in the Republic of Buryatia have the right to receive a land lot for individual housing development free of charge. However, such families living in the Tunkinsky National Park cannot exercise this right, because the land is federally owned and withdrawn from economic circulation. The value of these losses was:

$${AD}_{10}=295\;families*400\;sq.m.*0.14231\;thous.rub./sq.m.=16,792.58\;thous.rub.$$

Thus, the total amount of actual damage to the local residents living in the Tunkinsky National Park due to legal restrictions on the registration of land lots (*AD*_pop_) was 11,504.7 million rubles. Taking into account the potential amount of administrative fines and the value of the property to be demolished—16,462.4 million rubles.

The calculation of the amount of lost profits was made through the rate of return on invested capital (*i*). The key rate of the Bank of Russia in effect at the beginning of 2020 was used as the rate of return (6.25%). For the *t*-period during which no income could be received as a result of the diversion of funds, we took the period equal to the time since the legal restrictions were introduced, i.e., from 2011 to 2019 (9 years).

Based on the obtained values of actual damage to the population, we calculated the income lost as a result of the diversion of money for the reservation of funds for the purchase of land and construction (*LP*_1_), relocation (*LP*_2_), and fines (*LP*_3_):

$${LP}_{1}=1,076,539.46\;thous.rub.*({\left(1+0.0625\right)}^{9}-1)=781,223.94\;thous.rub.$$

$${LP}_{2}=8,212.5\;thous.rub.*({\left(1+0.0625\right)}^{9}-1)=5,959.65\;thous.rub.$$

$${LP}_{3}=535\;thous.rub.*({\left(1+0.0625\right)}^{9}-1)=388.24\;thous.rub.$$

Thus, according to our calculations, the bottom-line value of the losses of the population living in the Tunkinsky National Park due to legal restrictions on the registration of land dealings was 12.3 billion rubles, or 170.4 million USD (Table 4). Taking into account the potential amount of administrative fines and the value of property subject to demolition—17.2 billion rubles, or 239.2 million USD. This is 11 and 15 times the amount of own revenues of the Tunkinsky municipal district in 2011–2019.

**Table 4:** Total indicators of losses of the population living within the boundaries of Tunkinsky National Park as a result of legal restrictions on registration of land dealings.

	Indicators	Value, thous.rub.	Value, thous.USD^a^	Share in losses, %^b^
1	Potential capital costs of construction of real estate objects or infrastructure (*AD*′_1_), for demolition/relocation of which orders were issued	4950716,07	68639,83	- (28,7)
2	The amount of paid administrative fines for the lack of title documents for land lots (*AD*_2_)	535	7,42	0,004 (0,003)
3	Potential amount of administrative fines for the lack of title documents for land lots (*AD*′_2_)	7510	104,12	- (0,044)
4	Loss of personal income from personal subsidiary lots, (*AD*_4_)	162152,2	2248,18	1,319 (0,94)
5	Costs of forced relocation (*AD*_5_)	8212,5	113,86	0,067 (0,048)
6	Expenses for the purchase of land and construction to replace the lost property (*AD*_6_)	1076539,46	14925,82	8,758 (6,241)
7	The cost of full or partial loss of property due to relocation from protected areas (*AD*_8_)	10026683,27	139016,21	81,569 (58,126)
8	Material losses of the people due to inability to sell land lots (*AD*_9_)	213749,62	2963,56	1,739 (1,239)
9	Material losses of the people incurred as a result of the failure to provide social support (*AD*_10_)	16792,58	232,82	0,137 (0,097)
10	The total amount of actual damage (2+4+5+6+7+8+9)	11504700	159508,36	93,593 (4,529)
11	The total amount of actual damage taking into account the potential amount of administrative fines and the value of the property to be demolished (стр. 1+3+10)	16462400	228245,02	- (95,434)
12	The income lost as a result of the diversion of money for the reservation of funds for the purchase of land and construction (LP_1_)	781223,94	10831,38	6,355 (4,529)
13	The income lost as a result of the diversion of money for the reservation of funds for relocation	5959,65	82,63	0,048 (0,035)
14	The income lost as a result of the diversion of money for the reservation of funds for fines (LP_3_):	388,24	5,38	0,003 (0,002)
15	Lost profits (12+13+14)	787571,83	10919,39	6,407 (4,566)
16	The total value of the losses (10 + 15)	12292271,83	170427,75	100 (71,26)
17	The total value of the losses taking into account the potential amount of administrative fines and the value of property subject to demolition (11 + 15)	17249971,83	239164,41	- (100)

Source: Compiled by the authors.

^a^ Based on the average annual US dollar exchange rate for 2020 equal to 72.126 rubles.

^b^The parentheses in damages in view of the potential penalties and the cost of the property to be demolished.

More than 93% of the losses are attributable to the actual damage. In turn, the largest proportion of the actual damage consists of full or partial loss of property due to relocation beyond the borders of the national park.

## Conclusion

The assessment of social impacts of the creation and functioning of protected areas is increasingly becoming a common procedure for measuring the impact of conservation measures on local populations. Recognizing that the range of impacts is very wide and that there are still no generally accepted methodological tools for assessment, in this study we focused on the development of a methodology for quantifying the social impacts of protected areas arising from restrictions (infringements) on the ownership rights to land and property assets of local residents, as well as on the measures of their social support.

The methodological approach to the assessment of social impacts of protected areas developed by the authors is based on the consideration of the category of losses, which is understood as a sum-total of actual damage and lost profits of individuals (citizens) whose rights have been infringed. The assessment algorithm consists of three stages: systematization of social impacts on citizens, development of indicators and data collection, and calculation of actual damage to the population and lost profits. The assessment is based on the example of one of the largest national parks in Russia—Tunkinsky National Park, the area of which completely covers the territory of the Tunkinsky municipal district. There are 35 settlements with a population of over 20 thousand people within the boundaries of the park. The losses of the rural population due to legal restrictions on land registration are estimated at 12.3 billion rubles (170.4 million USD) and taking into account the potential amount of administrative fines—17.2 billion rubles (239.2 million USD) This is 11 and 15 times more than the amount of own revenues of the Tunkinsky municipal district generated during the period since the restrictions came to force in 2011.

The results prove the negative impact of the conservation policy on that part of the population in the Tunkinsky district who for nine years have not been able to register ownership rights to the land lots on which their houses and auxiliary buildings stand.

Starting in 2021, according to the amended Russian legislation, local residents have the right to privatize land in communities located in the park. In the long term, it can improve the quality of life of local people. However, the real picture of reducing the social impact of protected areas is still far from ideal, since there are still unresolved issues of land turnover in settlements with unspecified boundaries. The policy of improving land use for local residents requires updating the functional zoning of the National Park. It will allow to optimally combine the preservation of natural, historical, and cultural sites of the park with the economic use of the territory and the development of entrepreneurship and other opportunities for the local residents to earn a livelihood. Besides, in order to achieve the goals, continuous organizational and administrative work will be required both to register the rights of local residents to their land lots and to include the land with roads, bridges, power lines, pastures, and other sites and facilities that enable the smooth functioning of daily lives of people into the land of settlements. A partnership between the National Park and the local authorities seems to be inevitable, and they will work together to mitigate the effects of conservation policies on local communities.

We believe that this study contributes to the discussions on assessing potentially positive and negative impacts of measures aimed at developing protected areas that affect the rights of people and the quality of life of the community, as measured by various social and economic indicators.

Moreover, we assume that the methodology for estimating damages to local residents, which was tested using general indicators and open-source data for a specific national park in Russia, may be useful for measuring damages arising from restricting the rights of people to land in other national parks that have rural settlements with people living in them.

## Supporting information

S1 Table Share of unregistered land lots in the total number of lands lots of rural settlements of Tunkinsky district. (XLSX)

S2 Table Number of the local population and migration decrease of Tunkinsky district. (XLSX)

S3 Table Dynamics of the municipality’s own revenue of Tunkinsky district. (XLSX)
